# NMR-based metabolomics in real-time monitoring of treatment induced toxicity and cachexia in head and neck cancer: a method for early detection of high risk patients

**DOI:** 10.1007/s11306-019-1576-4

**Published:** 2019-08-16

**Authors:** Ł. Boguszewicz, A. Bieleń, J. Mrochem-Kwarciak, A. Skorupa, M. Ciszek, A. Heyda, A. Wygoda, A. Kotylak, K. Składowski, M. Sokół

**Affiliations:** 1 Department of Medical Physics, Maria Sklodowska-Curie Institute - Oncology Center Gliwice Branch, Wybrzeze Armii Krajowej 15, 44-101 Gliwice, Poland; 2 I Radiation And Clinical Oncology Department, Maria Sklodowska-Curie Institute - Oncology Center Gliwice Branch, Wybrzeze Armii Krajowej 15, Gliwice, 44-101 Poland; 3 Analytics and Clinical Biochemistry Department, Maria Sklodowska-Curie Institute - Oncology Center Gliwice Branch, Wybrzeze Armii Krajowej 15, Gliwice, 44-101 Poland

**Keywords:** MRS, NMR, Cancer, Radiotherapy, Chemotherapy, Cachexia

## Abstract

**Introduction:**

Nutritional treatment in head and neck squamous cell carcinoma cancer (HNSCC) patients undergoing radio-/chemo-radiotherapy (RT/CHRT) is complex and requires a multidisciplinary approach. In this study the real-time dynamic changes in serum metabolome during RT/CHRT in HNSCC patients were monitored using NMR-based metabolomics.

**Objectives:**

The main goal was to find the metabolic markers that could help prevent of acute radiation sequelae (ARS) escalation.

**Methods:**

170 HNSCC patients were treated radically with RT/CHRT. Blood samples were collected weekly, starting from the day before the treatment and stopping within the week after the RT/CHRT completion, resulting in a total number of 1328 samples. ^1^H NMR spectra were acquired on Bruker 400 MHz spectrometer at 310 K and analyzed using principal component analysis (PCA) and orthogonal partial least squares discriminant analysis (OPLS-DA). Additional statistical analyses were performed on the quantified metabolites.

**Results:**

PCA has detected a group of distinct outliers corresponding to ketone bodies (3HB, Ace, AceAce). These outliers were found to identify the individuals at high risk of weight loss, mainly by the 3HB changes, which was confirmed by the patients’ medical data. In the OPLS-DA models a transition from the lowest to the highest weight loss is seen, defining the metabolic time trajectories for the patients from the studied groups during RT/CHRT. 3HB is a relatively sensitive marker that allows earlier identification of the patients at higher risk of > 10% weight loss.

**Conclusion:**

Our findings indicate that metabolic alterations, characteristic for malnutrition or cachexia, can be detected already at the beginning of the treatment, making it possible to monitor the patients with a higher risk of weight loss.

**Electronic supplementary material:**

The online version of this article (10.1007/s11306-019-1576-4) contains supplementary material, which is available to authorized users.

## Introduction

Head and neck squamous cell carcinomas (HNSCC) are mainly located in larynx, pharynx and oral cavity, which play crucial roles in respiratory, nutritional, social and communicative functions. The standard organ preservation treatment method for HNSCC is sequential and/or concurrent radiotherapy (RT) and chemotherapy (CHT). However, it is associated with significant temporary or permanent toxic side effects in normal tissue and/or involved regions (acute radiation sequelae, ARS). The most common treatment induced toxicities are: mucositis (inflammation of the oral mucosa), xerostomia (sensation of dryness in the mouth), dysgeusia (altered or lack of taste), dysphagia (difficulty in swallowing) as well as radiation caries (tooth decay) and osteoradionecrosis (late adverse effect characterized by ischemic necrosis of the bone) (Siddiqui and Movsas 2017). These adverse effects not only impair the patients’ quality of life but can also lead to unplanned therapy interruptions. Such interruptions are a major problem affecting treatment outcome for many neoplasms, as increasingly shown in the literature (González Ferreira et al. 2015). In HNSCC a 1 day gap in radiotherapy could reduce the local control (time without any radiological and clinical signs of disease failure in place of primary occult) rate by 1.4%, while a gap of 1 week is associated with an absolute reduction in local control rates of 10–12% (Bese et al. 2007). In squamous cell carcinomas not only the duration of treatment break is of importance—also its timing is a crucial factor determining the chances of tumor cure (Herrmann et al. 1994). The detrimental effect of treatment interruptions increases as treatment progresses and ARS increases. As reveals from a large cohort study (Skladowski et al. 1994) a gap at the end of treatment may lead to a severe drop in a local control.

With the improvement of cancer therapy survival related to malignancy has improved, however in routine RT and CHT treatment monitoring there is still a need for sensitive and reproducible biomarkers predictive of a treatment toxicity. The molecular indicators of individual response to radiation would greatly improve monitoring of radiotherapy tolerance and allow early detection of ARS, its prevention and adequate supportive treatment. They could open a possibility for individualized and adaptive treatment programs based on appropriate protective measures.The HNSCC treatment induced toxicity has been extensively studied at both clinical (De Sanctis et al. 2016; Schindler et al. 2015) and metabolic/proteomic levels (Boguszewicz et al. 2016; Guerra et al. 2016; Jelonek et al. 2017; Roś-Mazurczyk et al. 2017; Widlak et al. 2016). Since the metabolite composition of blood plasma and serum is known to reflect the response of organisms to disease as well as the treatment-related and environmental factors (Trifonova et al. 2013), the patients susceptible to high ARS can be identified using NMR-based metabolomics (Boguszewicz et al. 2016) and/or mass spectrometry (Jelonek et al. 2017).

Widłak et al. showed that RT-induced changes observed at the level of serum proteome and lipidome reflect the most general response of the patient’s body to radiation, including inflammation and acute phase response (Widlak et al. 2016). The magnitude of the changes observed in the blood metabolome and proteome were found to correlate with the RT intensity as well as with the volume of side-irradiated normal tissue (Jelonek et al. 2017). Another mass spectrometry study by Roś-Mazurczyk et al. indicated a significant increase of 3-hydroxybutyrate (D(–)-3-hydroxybutyrate; β-hydroxybutyrate; 3HB) in the post-RT serum of HNSCC patients (Roś-Mazurczyk et al. 2017). Unfortunately, they did not correlate their findings with the patients’ clinical state.

3HB, acetone (Ace) and acetoacetate (AceAce) are collectively known as the ketone bodies. 3HB and AceAce are produced in considerable quantities in the liver under metabolic conditions associated with a high rate of fatty acid oxidation. Acetoacetate continually undergoes spontaneous decarboxylation to yield acetone. These three metabolites: 3HB, Ace and AceAce serve as a circulating energy source for tissues in times of fasting (Murray et al. 2009; Newman and Verdin 2014). In humans, their basal serum levels are extremely low in normal condition, being almost beyond the NMR detection range. For instance, the β-hydroxybutyrate levels are in the low micromolar range, but fasting leads to their rise to a few hundred micromoles after several hours of fasting, and to the values of several millimoles after several days of fasting (Cahill 2006; Murray et al. 2009; Newman and Verdin 2014). 3HB is released into the bloodstream during fasting and reflects ketosis. The ketone body ratio (the ratio of 3HB to acetoacetate) is normally 1:1, rising to 6:1 with prolonged fasting, and up to 10:1 in pathological ketosis. Thus, 3HB is a very representative ketone during ketosis (Chiu et al. 2002), which makes it a valuable biomarker.

HNSCC patients are often malnourished due to tumor involvement of the swallowing structures, but treatment induced swallowing dysfunction, leading to prolonged fasting, has been reported in 30–50% of HNSCC patients treated with non-surgical regimens (Schindler et al. 2015). Thus, a nutritional intervention in head and neck cancer patients undergoing radio- and chemo-radiotherapy is necessary, but the nutritional treatment of these patients is complex and requires a multidisciplinary approach. Our recent findings concerning during the treatment monitoring of the head and neck cancer patients suggest that the increased serum 3HB levels may reflect the intensified ARS and treatment induced dysphagia in HNSCC patients treated with RT/CHRT (Boguszewicz et al. 2017a, b). In our previous study (Boguszewicz et al. 2016) related to the early RT/CHRT treatment-related toxicities (in the mentioned work we compared the NMR serum metabolic profiles acquired before and after the treatment) we also observed the increased levels of 3HB in HNSCC patients with high ARS, however this increase was not statistically significant when compared to the patients with low ARS.

In this study we decided to investigate the real-time dynamic changes in the serum metabolome during RT or CHRT in HNSCC patients. The aim was to identify the ARS escalation biomarkers which could be used to prevent the consequences of ARS, thus to find the metabolic markers predicting the adverse treatment related effects before their onset. The molecular monitoring of ARS is expected to improve the overall patients’ quality of life, their performance status as well as to reduce the risk of unplanned gaps during the treatment.

## Materials and methods

### Characteristics of patient groups

The study was approved by the Ethics Committee and the informed consent of participants was obtained.

The studied group consisted of 170 HNSCC patients treated oncologically in the 1st Radiation and Clinical Oncology Department of MSC Memorial Institute in Gliwice. There were 136 men and 34 women, all Caucasians, at median age of 59.5 (22–80 years). Cancer was diagnosed in fifteen anatomical sub-regions of the head and neck (as listed in Supplementary Material), with distinction of four major sites: nasopharynx (11 patients), oropharynx (50 patients), hypopharynx (22 patients), larynx (77 patients) and other (10 patients).

The primary tumor stage was scored according to the TNM scale. Cancer staging describes how far cancer has spread anatomically. The stage generally takes into account tumor size and tumor invasion of an adjacent organ (T stage), how many regional lymph nodes are involved (N stage) and distant metastases presence (M stage). The clinical stage is based on total available information obtained about tumor from the physical examination, blood tests, radiologic examination, biopsy, and endoscopy.

The larynx group was divided into low stage disease patients (T1N0M0 and T2N0M0—37 patients) and high stage (other—40 patients), because of different radiotherapy range (low stage disease smaller treatment plan volume) and the negligible weight losses (< 5%) for the majority of the patients of this group during the treatment.

The patients involved in the current study were clinically staged before the treatment into the following groups: T1 (11.2%), T2 (33.5%), T3 (30.5%), T4 (21.5%), T0 N + (3.5%), N + mean locoregional disease (54.7%). There were no patients with metastases (M0 = 100%).

All patients were treated with radical intent, 37.5% followed RT and 62.5%—CHRT. CHRT was performed as an induction treatment (iCHRT 1–3 cycles) in 36% of patients (Docetaxel, Cisplatin, 5-Fluorouracil—TPF regimen or Cisplatin, 5-Fluorouracil—PF regimen) or/and concurrent treatment (cCHRT) in 26.5% patients, using Cisplatin, fractionated from the first day of RT, with 21 days interval (2–3 cycles). Overall RT doses varied between 51 and 72 Gy. The patients with very advanced cancer (high TNM stages) received more aggressive treatment and their radiation treatment volumes were expanded.

### Evaluation of ARS

ARS was evaluated using MultiParametric Monitoring (MPM)—an original evaluation system designed by the study investigators (Hajduk et al. 2012; Składowski et al. 2012). This system is based on the existing rules of the Common Toxicity Criteria of Adverse Event (CTCAE) scale, but in contrast to CTCAE it does not analyze individual symptoms separately, but assesses all symptoms related to the irradiated fields and affected functions collectively. The evaluation of ARS using MPM was done as described by Boguszewicz et al. (2016). The core elements of ARS evaluation are: medical examination, endoscopic examination and laboratory blood tests.

### Serum samples collection

The overnight fasting blood samples from the peripheral vein were collected weekly, starting from the day before the treatment and stopping within the week after the RT/CHRT completion, resulting in a total number of 1328 samples. The collected samples were divided into two aliquots, one of which was used for the laboratory blood tests (ARS evaluation), the other was used for the ^1^H NMR spectroscopic analysis (metabolic profiling of ARS). The samples were incubated for 30 min at room temperature and then centrifuged (1000×*g*, 10 min) to remove a clot, and stored frozen at − 80 °C until NMR measurements are performed.

### Design of the study

**Fig. 1:**
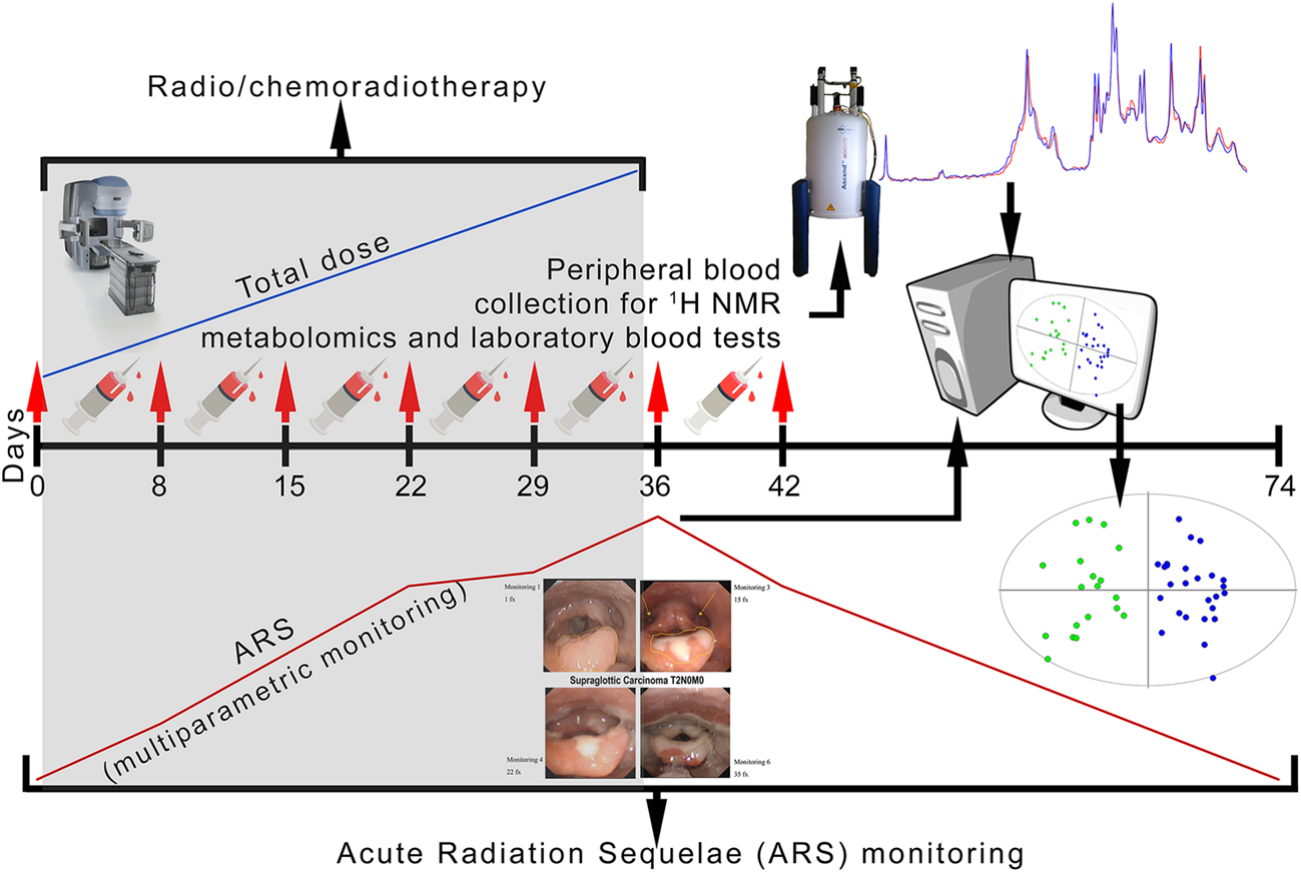
The design of the study—an exemplary course. ARS monitoring starts at the day before the treatment and is continued until the resolution of all ARS symptoms. The blood samples for the NMR-based metabolomics study as well as the standard laboratory blood tests are collected at the same time intervals as the clinical monitoring. Reproduced with permission from Boguszewicz et al. (2016). Copyright 2016

The scheme of the study is presented in Fig. 1—the exemplary total dose and ARS curves are shown in order to visualize the idea of the data collection procedures. The fractionation and total doses vary between the treatments modalities. The highest ARS is observed usually during the second half of RT/CHRT.

### Sample preparation for NMR spectroscopy

The samples for the metabolomics experiment were prepared according to the modified Bruker protocol described previously (Boguszewicz et al. 2016). The protocol involves the two-step thawing (at 4 °C and at room temperature) and using phosphate buffer (pH 7.4) with D_2_O and TSP. The aliquots of 600 µl of the solution are transferred into 5 mm Wilmad WG-1235-7 NMR tubes (Wilmad Labglass, USA) and kept at 4 °C until the NMR analysis.

### Measurement protocols

^1^H NMR spectra were acquired on a Bruker 400 MHz Avance III spectrometer (Bruker Biospin, Rheinstetten, Germany) equipped with a 5 mm PABBI probe. The quality control tests were performed at every measurement day. The NMR probe tuning and matching, shimming, determination of the transmitter offset value for the water pulse presaturation and 90° pulse adjustments were always made for each sample. The receiver gain was set to 90.5 and the temperature to 310 K for all experiments.

Four different ^1^H NMR experiments were performed for each serum sample:

- NOESY (Nuclear Overhauser effect spectroscopy)—to obtain an overview of all types of molecules.
- CPMG (Carr-Purcell-Meiboom-Gill)—to get information on only low molecular weight metabolites.
- DIFF (diffusion edited)—to detect mainly macromolecular signals.
- Two dimensional (2D) JRES (J-resolved)—to visualize scalar couplings and improve metabolite identification, while 1D projections of J-resolved spectra were used in data analyses. 1D J-resolved projections show signals from small metabolites, similar to CPMG, but due to homodecoupling each signal appears as a singlet. For any particular ^1^H resonance, data for all J-couplings appear in the projected 1D JRES spectrum at the same ppm. Removal of J-coupling information from 1D ^1^H spectra in this way reduces the overlap of resonance peaks from different metabolites, and allows more accurate metabolite quantifications. Therefore, such projections are easier to analyze and quantify.

The total number of acquired NMR spectra was 5312 (4 spectra per each of 1328 samples).

The pulse sequence parameters are given in Supplementary Material.

### Spectra post-processing

All 1D spectra were processed with a line broadening of 0.3 Hz and automatically phase corrected (in Topspin software from Bruker Biospin), referenced to the methyl doublet of alanine at 1.5 ppm and bucketed over the region 9.0–0.5 ppm with the bucket width set to 0.002 ppm using AMIX software (Bruker Biospin). The spectrum region of water (5.15–4.38 ppm, d = 0.77 ppm) was removed from the analysis in order to prevent variation in each sample. No normalization was applied. Our previous results show that strict adherence to the sample preparation protocol and the measurement protocol provides very stable and reproducible results allowing for biological interpretations (Boguszewicz et al. 2016, 2017a, b).

### Metabolite identification

The metabolites were identified by comparing their chemical shifts with those for the standard compounds from Chenomx NMR Suite Professional database (Chenomx Inc., Edmonton, Canada). In case of assignment ambiguity spectral identification was supported with multiplicity and scalar couplings information from the 2D J-resolved spectra as well as Human Metabolome Database and available literature.

### Metabolite quantification

1D projections of the J-resolved spectra were used for quantification of the low molecular weight metabolites, while the lipid signals were quantified based on the diffusion edited spectra. The peaks in the 1D spectra were integrated by area in AMIX software (Bruker, Biospin). The peak integrals were measured in a spectral region of 0.012 ppm (low molecular weight metabolites) and of 0.12 ppm (lipids) centered around a particular peak.

### Data analysis

The data analysis was carried out using SIMCA-P + (Umetrics, v. 14) software. The multivariate projection techniques (MPT) of principal component analysis (PCA) and supervised orthogonal partial least squares discriminant analysis, OPLS-DA—were applied.

The MPT results are presented graphically in two types of the plots:

- The scores plot—where each point represents one spectrum projected on a reduced plane spread on the directions of the highest variance (PCA) or the directions representing the between class variation (OPLS-DA).
- The loadings plot—where each point represents one spectral point (or one spectral bucket). The point distance from the plot center corresponds to the contribution of a particular spectroscopic signal to the principal component (PCA) or displays the relationship between the spectral points (spectroscopic signals) and the class separation (position of the point in the scores plot) in OPLS-DA.

The statistical analyses (Mann–Whitney *U* test, MWU) and Kruskal–Wallis ANOVA (KW ANOVA) were performed with Statistica software (Statsoft, v. 12).

## Results

**Fig. 2:**
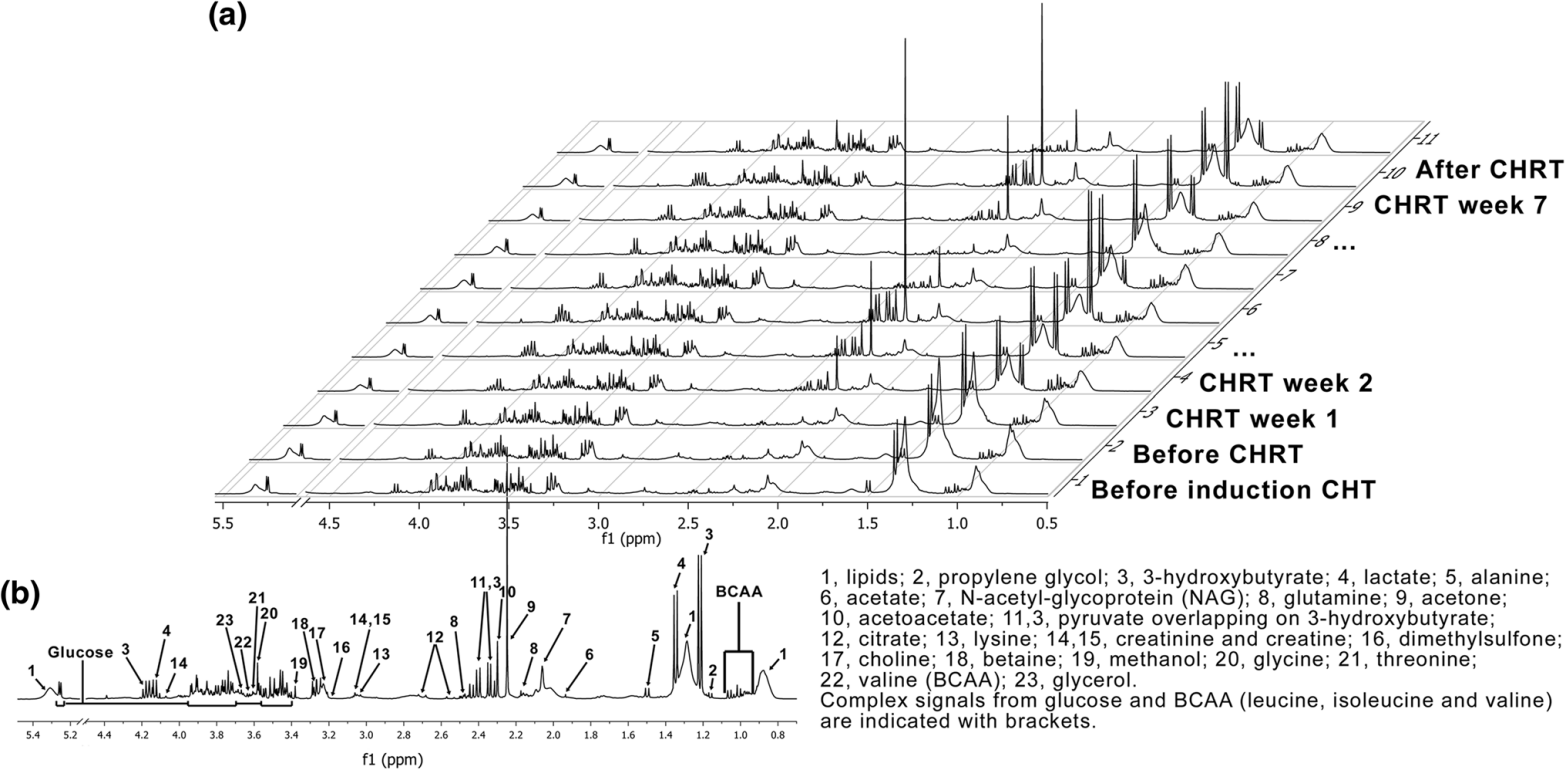
400 MHz ^1^H NMR CPMG stacked spectra of the HNSCC patient treated with induction CHT followed by a concurrent CHRT (**a**). The main detected metabolites are indicated in (**b**)

The 400 MHz ^1^H NMR CPMG stacked spectra of the chosen HNSCC patient treated with induction CHRT followed by a concurrent CHRT are shown in Fig. 2. The spectra were acquired a day before the first cycle of induction CHRT, a day before the start of CHRT, and then in weekly intervals during the CHRT course. The last two spectra were acquired four and eleven days after the completion of CHRT, respectively. The main detected metabolites are indicated.

The most explicit observation resulting from the analysis of Fig. 2 is a huge, two-stage increase in the signals from 3HB, Ace and AceAce—first, between the second and fifth week, and then after the seventh week of CHRT. In our study, the detailed examination of the patients’ clinical data revealed that the increase of 3HB correlates with the episodes of severe functional and morphological ARS (strong pain and severe dysphagia, fluid diet only or refusal to eat/drink at all). These patients received intravenous rehydration and drug supplementation to relieve the ARS symptoms resulting in temporary lowering of the 3HB signals. In case of three patients the intensification of ARS caused an unplanned gap in the treatment schedule. Two of these patients died, one due to local failure and one due to locoregional failure and distant disease.

Based on these findings we decided to investigate the correlations between 3HB and lipids as well as other parameters from MPM (the patients’ clinical data and laboratory blood tests). Because the presence of 3HB in serum is an indication of ketosis, we decided to follow the changes in the integral of the 3HB doublet at δ 1.22 ppm, which corresponds to a CH_3_ group. This signal does not overlap with the signals from other metabolites and, thus, it is easy to quantify with a good precision, especially in 1D JRES spectra. Moreover, 3HB, the most abundant circulating ketone body, is chemically stable and, thus, is less likely to degrade spontaneously into acetone than acetoacetate (Newman and Verdin 2014).

**Fig. 3:**
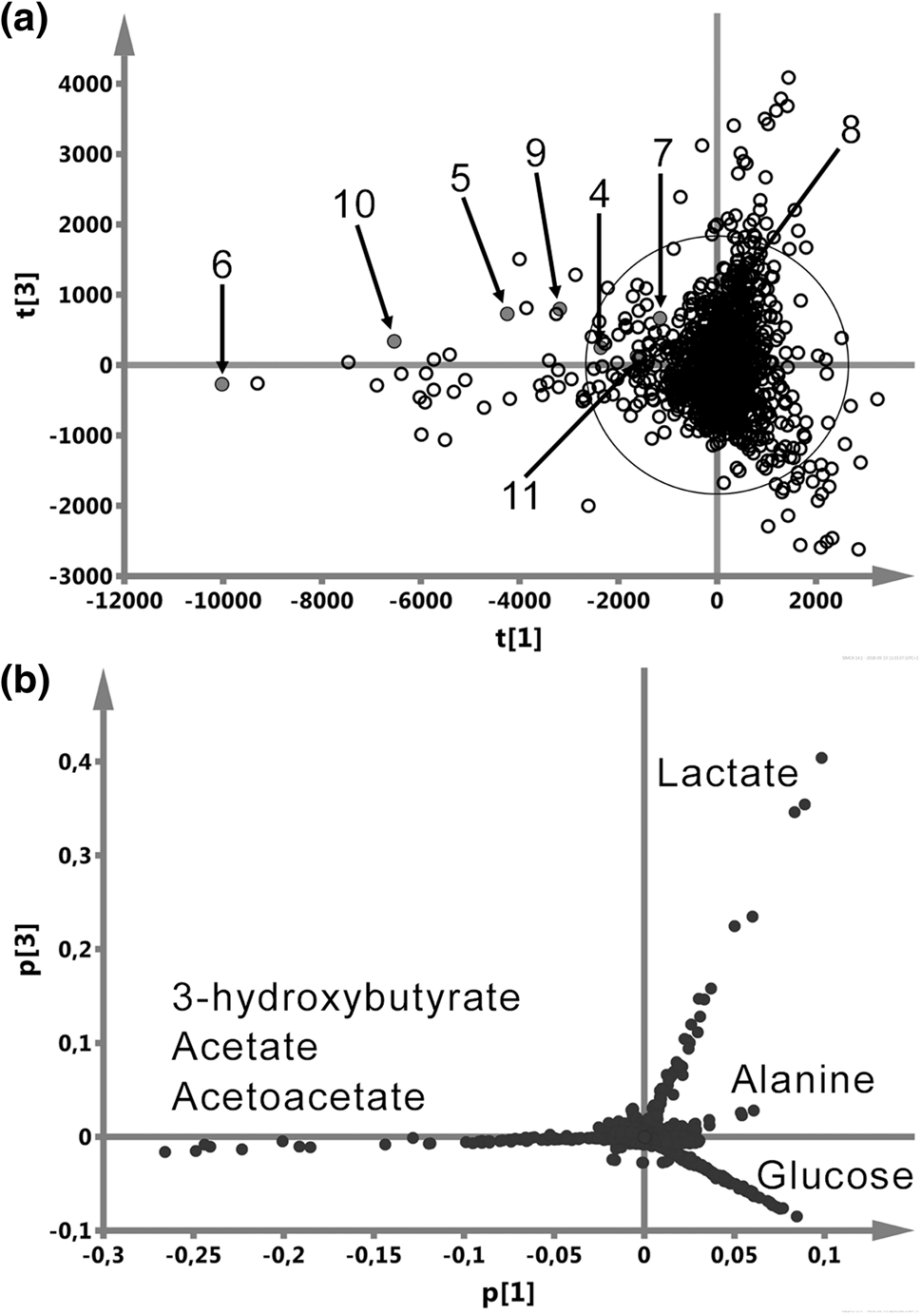
PCA scores (**a**) and loadings (**b**) plots show the distinct outliers due to 3HB, Ace, AceAce along p[1] as well as the lactate (Lac) and glucose (Gluc) signals along p[3]. The numbers in (**a**) denote the consecutive spectra of the chosen HNSCC patient (the same as in Fig. 2), acquired before (the points are hidden in the central cloud of points), during (4–9) and after (10, 11) the CHRT course

Unsupervised PCA was chosen to visualize the directions of the largest variation in the spectroscopic data and to detect the outlying spectra. Due to a large variation in the lipid signals contributing to the first few components of the PCA model built on the CPMG spectra, the 1D JRES spectra were used. Figure 3 shows the PCA scores (a) and loadings (b) plots with the distinct outliers due to 3HB, Ace and AceAce along p[1] as well as to lactate (Lac) and glucose (Gluc) along p[3]. The points denoted in Fig. 3a by numbers indicate the consecutive spectra acquired for the same HNSCC patient as in Fig. 2 before (the points are hidden within the central cloud), during (4–9) and after (10, 11) the CHRT course.

This patient’s baseline levels of the main nutritional and inflammatory laboratory markers (as weight, percentage weight loss, BMI as well as the albumin, prealbumin and CRP levels) are collected in Table 1. The values falling outside the reference range are marked with two asterisks (**). The integral values of the 3HB signal at δ 1.22 ppm were normalized in the 0–1 range to make the comparisons easier.

**Table 1:** The changes in 3-hydroxybutyrate as well as other clinical parameters during the treatment course for the patient denoted in Figs. 2 and 3

Spectrum no.^a^	Treatment course	Integral intensity of the 3HB signal at 1.22 ppm (normalized^b^)	Weight (kg)	Weight loss (%)	BMI (kg/m^2^)	ALB (g/l)	PreALB (g/l)	CRP (mg/l)
1	Before induction CHT	0.000	73	0	25	47.9	0.303	1.15
2	Before CHRT	0.004	76	− 4.1	26	42.2	0.342	4.19^c^
3	CHRT week 1	0.011	76	− 4.1	26	44.7	0.295	1.87
4	CHRT week 2	0.317	70.5	3.4	24	50.3	0.367	1.04
5	CHRT week 3	0.544	69	5.5	23.6	46.4	0.423	2.85
6	CHRT week 4	1.000	64.8	11.2	22.2	46.3	0.177^c^	17.6^c^
7	CHRT week 5	0.181	61.6	15.6	21.1	46.5	0.423	1.05
8	CHRT week 6	0.032	60.7	16.8	20.8	37.3	0.182^c^	36.7^c^
9	CHRT week 7	0.317	57.1	21.8	19.5	37.9	0.141^c^	50.3^c^
10	Four days after CHRT	0.701	56.3	22.9	19.3	38.7	0.115^c^	81.1^c^
11	Eleven days after CHRT	0.192	56.3	22.9	19.3	38.1	0.157^c^	18.3^c^

^a^The numbering of the spectra corresponds to the numbers in Figs. 2 and 3

^b^To facilitate the detection of changes in the 3HB, the integral intensities have been normalized to the highest value (= 1)

^c^These values are below the reference range for body mass index (BMI), albumin (ALB), prealbumin (PreALB) and above the reference range for CRP

**Fig. 4:**
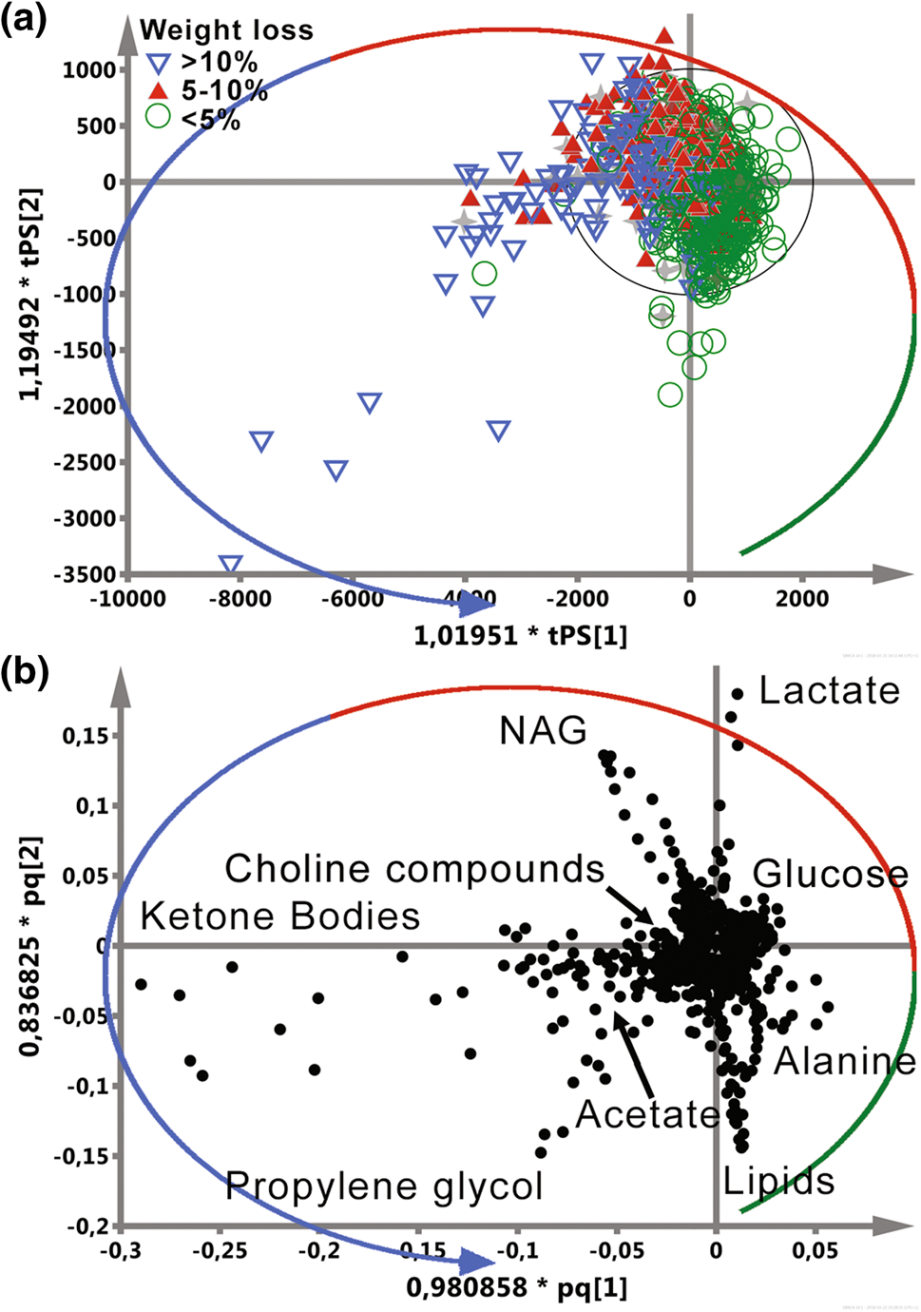
OPLS-DA analysis of the 1D 1H JRES NMR serum spectra. The colors and shapes of the points identify the patient’s percentage weight loss on the day of acquiring a blood sample for the analysis. The scores plot (**a**) shows the anticlockwise transition from < 5 to > 10% weight loss during the RT/CHRT course. The metabolic profiles correlated with the percentage weight loss are identified based on the loadings plot (**b**)

Supervised OPLS-DA was chosen to investigate the correlations between the metabolic profile and weight loss as well as to possibly identify the patients with high risk of significant weight loss during the treatment (Fig. 4). The literature discussing the treatment induced weight loss and cachexia in head and neck cancer patients is sparse and there is no consistency for classification of the treatment induced cachexia. Based on the results published by Cacicedo et al. (2014), Fearon et al. (2011) and Wallengren et al. (2013) we decided to distinguish three groups of the percentage weight loss: below 5%, between 5–10 and above 10%. The group sizes (with a distinction between the individual tumor sites) are shown in Table 2.

**Table 2:** The number of patients who achieved a given percentage weight loss during the RT/CHRT treatment with a distinction between the individual tumor sites

Tumor site	Weight loss			
	Number of patients			
	Below 5%	Between 5 and 10%	Above 10%	Incomplete weight record
Oropharynx	8	11	29	2
Hypopharynx	4	10	8	
Nasopharynx			11	
Larynx (high stage)	6	16	18	
Larynx (low stage)	23	9	5	
Other	1	5	4	
Sum	42	51	75	2

Figure 4a shows the OPLS-DA predicted scores plot where each point represents a single NMR spectrum of blood serum and its color and shape encode the actual (at the blood sample collection time) percentage weight loss class. The class names are chosen to distinguish between assigning a point (a spectrum) to a class— < 5%, 5–10% and > 10%—and a patient (based on the highest weight loss, as in Table 2) to a class. The class assignment to a single spectrum allows for tracking the dynamic changes in the metabolic profile in relation to the weight loss. The NoID samples (no weight record for a particular monitoring time-point during the RT/CHRT course) are projected onto the OPLS-DA plane. Though the percentage weight loss classes are not well separated, but a distinct transition from the lowest to the highest weight loss is clearly visible allowing for investigation of the time trajectories for the individual patients. The corresponding loadings plot (Fig. 4b) displays a relationship between the chemical shifts of the NMR spectrum and the percentage weight loss classes. Analyzing the plot anticlockwise (according to the transition observed in Fig. 4a), it is possible to identify the sequence of the metabolic events caused by ARS progressing which result in the weight loss. The changes in the metabolic profile start with the increased signals due to lipids (0.9 and 1.3 ppm) and phospholipids (3.2 ppm). Then, an increase of alanine (Ala) (1.5 ppm) is observed close to the 3 o’clock position. At the 12 o’clock position there is a decrease of lipids/phospholipids and increase of lactate (Lac) at 1.33 ppm. Moving further anticlockwise, there is an increase of N-acetyl-glycoprotein (NAG) at 2.07 ppm followed by a massive increase in the ketone bodies (3HB, Ace and AceAce) at the 8 o’clock position corresponding to the highest percentage weight loss. Finally, at the 7 o’clock position the increase of propylene glycol concentration takes place.

The statistical significance of the observed metabolic changes was tested using KW Anova test and the results, showing the differences in the OPLS-DA identified serum metabolites according to the percentage weight loss, are presented in Table 3. The percentage weight loss groups (< 5%, 5–10% and > 10%) are distinguishable by the statistically significant elevated concentrations of the ketone bodies (3HB, acetone and acetoacetate), choline and NAG as well as by significantly lowered concentrations of alanine. Additionally, a markedly elevated propylene glycol is observed. There are no statistically important differences in the signals due to lipids and acetate, while the glucose levels differentiate only < 5% and > 10% weight loss groups.

**Table 3:** The results from Kruskal–Wallis ANOVA showing the statistical differences in the serum metabolites according to the percentage weight loss in head and neck cancer patients

Metabolites	Weight loss groups		
		5–10%	>10%
↑ 3HB	< 5%	0.000	0.000
	5–10%		0.000
↑ Acetone	< 5%	0.000	0.000
	5–10%		0.000
↑ Acetoacetate	< 5%	0.000	0.000
	5–10%		0.000
Lipids	< 5%	0.806	1
	5–10%		1
↑ Choline	< 5%	0.000	0.000
	5–10%		0.000
↓ Alanine	< 5%	0.000	0.000
	5–10%		0.001
↑ Lactate	< 5%	0.933	1
	5–10%		1
↑ NAG	< 5%	0.000	0.000
	5–10%		0.003
↑ Propylene glycol	< 5%	1	0.000
	5–10%		0.000
↓ Glucose	< 5%	0.341	0.042
	5–10%		0.911
↑ Acetate	< 5%	1	1
	5–10%		1

↑—Positively correlated with weight loss

↓—Negatively correlated with weight loss

**Fig. 5:**
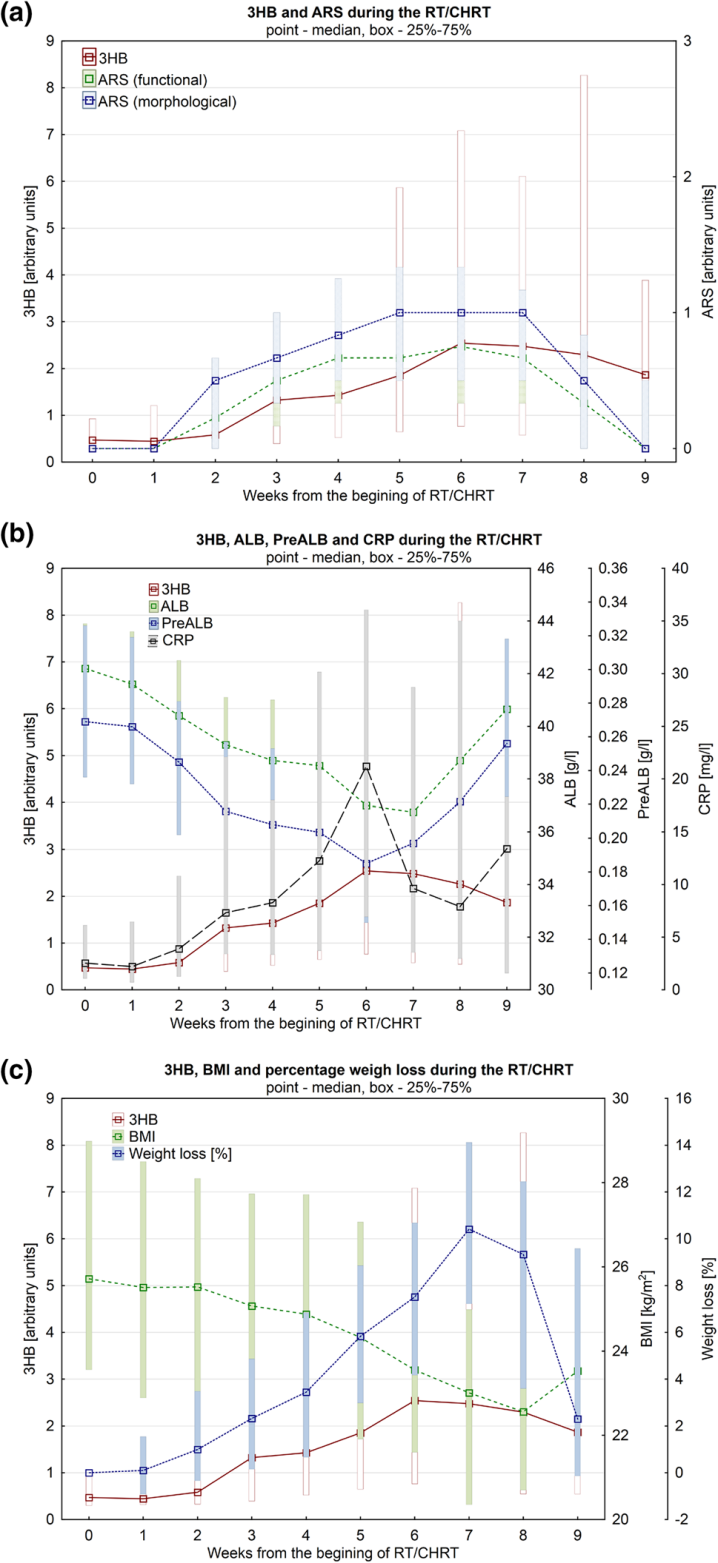
The changes in 3-hydroxybutyrate, ARS, CRP and the clinical parameters reflecting the patients’ nutritional status (albumin, prealbumin, BMI and percentage weight loss) during the RT/CHRT (Color figure online)

ARS was evaluated and scored in numerical values and, thus, it was possible to correlate the real-time changes in the ARS and 3HB levels (Fig. 5a). Similar plots were created to compare the real-time changes in 3HB versus ALB, PreALB and CRP (Fig. 5b) as well as in 3HB versus BMI and the percentage weight loss (Fig. 5c).

**Fig. 6:**
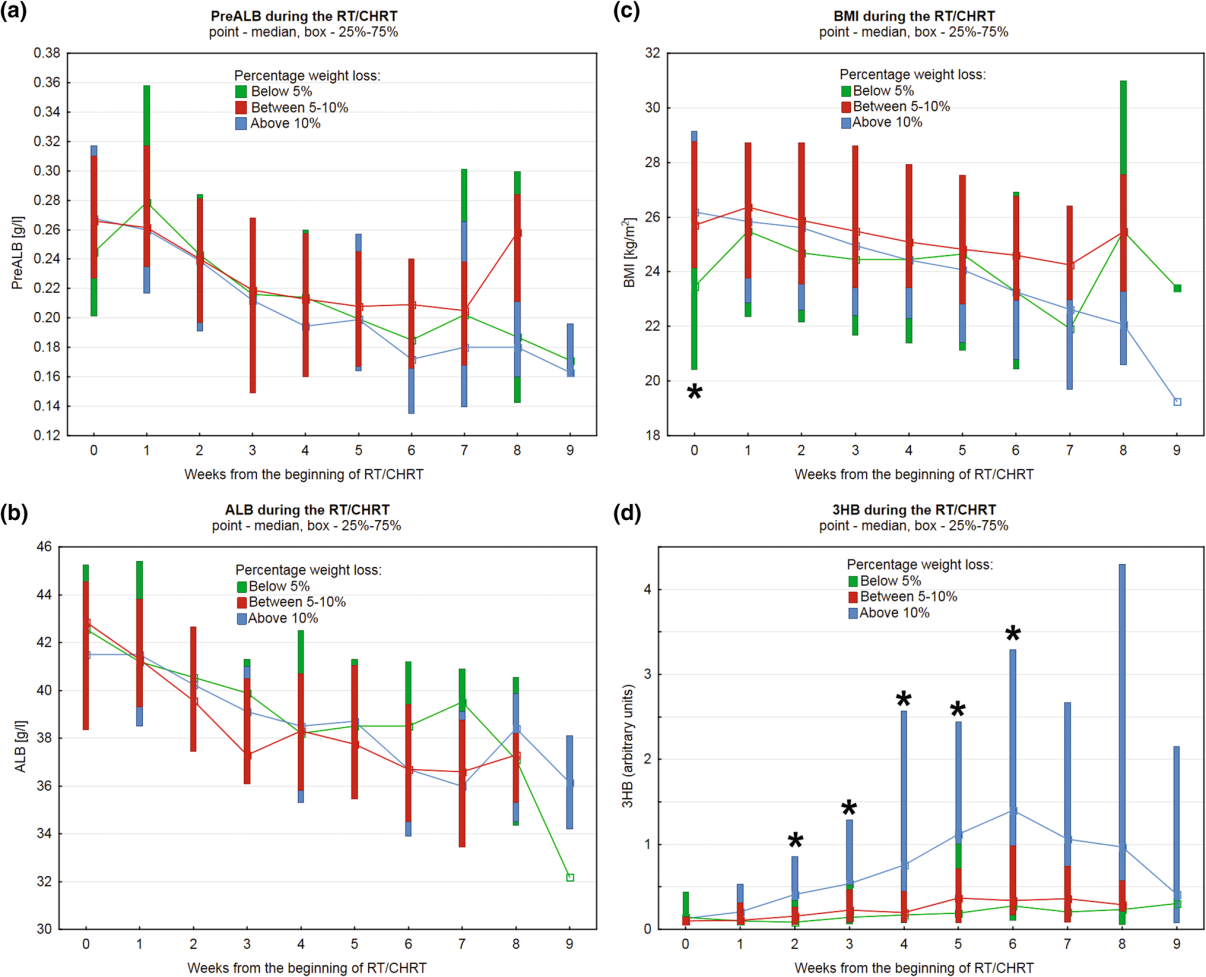
The changes in PreALB (**a**), ALB (**b**), BMI (**c**) and 3HB (**d**) during the RT/CHRT for the three percentage weight loss groups: below 5% (green), between 5-10% (red) and above 10% (blue). A clear separation of the above 10% group is seen and results from the 3-hydroxybutyrate (3HB) intensity variations. The statistically significant differences according to KW Anova are denoted with *. The patients with the lowest weight loss (green) start the anticancer treatment with significantly lower BMIs. However, because they receive the supportive treatment, their BMI values are seen to normalize within a week. The drop of the 3HB values after the 6th week in the above 10% group (blue) is due to the intensified supportive care aimed to stop further weight loss (Color figure online)

In order to check whether the initial (at the beginning of RT/CHRT) nutritional status of the patients—expressed in the ALB, PreALB and BMI values (Fig. 6a–c) as well as by the 3HB levels (Fig. 6d)—has an impact on the patients’ subsequent loss of body weight during the treatment, each patient was assigned to one of the three groups corresponding to their total percentage weight loss during treatment: below 5%, between 5–10 and above 10%. The Larynx (low stage) group was excluded from this analysis. The number of patients in each group (with a distinction between the individual tumor sites) is shown in Table 2. Such approach shows no statistical differences between the studied groups, while the patients with the lowest weight loss (below 5%) start the treatment with a significantly lower BMI values. Thus, the intensity variation of the 3HB signal is a relatively sensitive marker (statistically significant increase is observed already in the second week of treatment) identifying the patients at higher risk of > 10% weight loss. We also observed that the higher initial BMI (above 24 kg/m^2^) is connected with the higher risk of significant weight loss during the treatment (Fig. 6c). In the studied group (a total of 170 subjects), only six patients entered the RT/CHRT treatment with the nutritional clinical parameters lower than the reference values: one with BMI = 16.35 kg/m^2^ (ALB and PreALB within the normal reference range), four with ALB < 34 mg/ml, but with the normal/overweight BMI (two of them with PreALB below the normal reference range < 0.19 mg/ml) and one with PreALB = 0.128 mg/ml but with the normal BMI and ALB. The median initial BMI, ALB and PreALB values were 25.7 kg/m^2^, 42.2 mg/ml and 0.269 mg/ml, respectively.

In order to assess the influence of heterogeneity of the studied group on the 3HB concentrations we performed a careful verification of the possible factors (e.g. age, gender, tumor stage and site) using ANOVA and MWU tests as well as Spearman’s correlation coefficients. It was found that the integral values of the 3HB signal at 1.22 ppm correlate with the TNM overall stage (r = 0.31, p < 0.05) and lymph node (N) stages (r = 0.33, p < 0.05) while a correlation with primary tumor stage (T) is not statistically significant (r < 0.3, p = 0.065).

## Discussion

Radiation therapy and chemotherapy, despite being a successful treatment for cancer, cause additional adverse events, like acute radiation sequelae, systemic and psychological disturbances. These toxic effects together with the patient’s general condition (nutritional status, distress and pain level) and individual radio and chemosensitivity are the key factors influencing the effect of anticancer therapy, mean locoregional control rate, side effects and overall patients quality of life after all. Identifying unfavorable factors, before or at the very beginning of the treatment, would be important in minimizing the incidence of treatment complications, but first, the clinical and molecular markers of the adverse events must be precisely determined.

In this study the dynamic systemic response of the HNSCC patients to RT/CHRT was investigated during the treatment by ^1^H NMR metabolic phenotyping of serum and presented by PCA and OPLS-DA. The results of such combination of metabolic and clinical approaches strongly supports a possibility of using the ^1^H NMR serum metabolomics as the technique complementary with other clinical and laboratory tests useful in the patient alerting system. To the best of our knowledge, this is the first report on a real-time NMR metabolomic monitoring of the RT/CHRT induced molecular changes in blood serum and their correlation with the clinical parameters in a large group of HNSCC patients. The results presented here are a part of an ongoing grant project funded by the National Science Centre of Poland and mainly concern the identification and analysis of the outlying cases characterized by excessively high metabolic alterations. This is the preliminary stage preceding attempting to the insight into the subtle changes in the metabolic profile.

The PCA analysis applied to the ^1^H NMR serum data (Fig. 3) resulted in detection of a group of the distinct outliers—they are mainly due to the ketone bodies (3HB, Ace, AceAce), lactate and glucose. These outliers identify the individuals at high-risk of weight loss (Table 1)—mainly by the 3HB changes, and this observation is confirmed by the patients’ medical data.

In the OPLS-DA models a transition from the lowest to the highest weight loss is seen, defining the metabolic time trajectories for the patients from the studied groups during RT/CHRT (Fig. 4) and also showing 3HB to be an important indicator of the weight loss (a more in-depth analysis of these time trajectories is provided later). The increase of 3HB correlates with the episodes of the severe functional and morphological ARS (Fig. 5a) as well as with the TNM and lymph node stages. Moreover, there is a very good correlation (except one time-point—week 6) between 3HB and CRP (Fig. 5b). Though other clinical markers—ALB and PreALB (Fig. 5b)—also change during the therapy (inversely proportionally to 3HB), however, it is worth noting that the ALB and PreALB medians remain in the normal range, and the same is true for BMI—its lowering (with the simultaneous increase in 3HB and the percentage weight loss) is shown in Fig. 5c.

Because the changes in the 3HB serum level are correlated with the ARS-score and the clinical parameters (albumin, prealbumin, BMI and percentage weight loss) reflecting the patients’ treatment related nutritional deficiencies, 3HB may be considered as an indicator of early nutritional disturbances in HNSCC patients during the RT/CHRT treatment.

Anthropometric parameters, such as significant weight loss, and analytical parameters, such as albumin and prealbumin assessed together with CRP, are often used to get information about the nutritional status. But because many extrinsic and intrinsic factors affect these biomarkers (like infections, liver diseases, renal, dehydration, anasarca, etc.) (Virizuela et al. 2017), the interpretation of their changes is difficult. Such subacute or chronic condition of a complex nature, in which variable combinations of nutritional imbalance and inflammatory processes are responsible for modification of the body composition (reduction of muscle and/or fat mass) and alteration of the organ functions (immune, muscle and cognitive deficits) is defined as malnutrition (Soeters et al. 2008). Weight loss and malnutrition tend to develop particularly in patients in whom the segments of the gastrointestinal tract are subjected to irradiation.

As reveals from the analysis of Fig. 6, the PreALB, ALB and BMI changes detected during the RT/CHRT treatment are not due to the patients’ nutritional status before the anticancer treatment. All patients received adequate medical supportive care, which is reflected in BMI and the PreALB level normalization in the first week of the treatment in the group of patients with the lowest percentage loss of body weight (< 5%). As seen in Fig. 6 nearly all patients start the treatment with the normal nutritional clinical parameters, and more importantly, we note that the higher initial BMI (above 24 kg/m^2^) is connected with the higher risk of significant weight loss during the treatment (Fig. 6c).

We found that a nutritional deficiency and cachexia are associated with the escalation of ARS (as documented in Fig. 5), what is presumably a consequence of the primary cancer stage and disease extend (larger radiotherapy fields—higher ARS escalation). The relationship between the treatment intensity and the adverse events, like weight loss and malnutrition, is well documented in the literature (Couch et al. 2015; Unal et al. 2013), and confirmed in our results as well (Fig. 5, Table 2). However, personal radiosensitivity as well as the patient’s ability to deal with distress, with inflammatory process and tumor lysis may also be of great importance. These factors are hardly measurable, but we observed a significant weight loss (> 10%) also in a small group of patients with a relatively low ARS.

3HB may be considered as a convenient prognostic biomarker, because its concentration increases already in the first hours of significantly reduced food intake. At the same time, there is also a decrease in PreALB and, to a lesser extent, in ALB (which results from a difference in the protein half-lives: 18 to 20 days for albumin and ca. 2 days for prealbumin) and BMI, but these values remain, in most cases, still in the reference range. This apparent correctness may weaken the diagnostic vigilance and delay the reaction (rehydration, change to a mixed diet, parenteral nutrition) up to the moment when severe malnutrition and/or dehydration appear, as it did in our study. Moreover, with the introduction of supportive treatment the levels of 3HB, PreALB, CRP may normalize rapidly, falsely suggesting the adverse changes are stopped. However, in order to stop cachexia and to improve the patients’ performance status, a proper timing of nutritional intervention (when and how) is of fundamental importance.

Cachexia is strongly correlated with a poor quality of life, reduced tolerance of treatment and a shorter survival in many types of cancer including HNSCC (Aversa et al. 2017; Couch et al. 2015). However, a simple quantitative assessment of body weight can be misleading, because it does not provide the information on the body qualitative composition or the patient’s hydration status (Sukkar 2012). Moreover, the decrease in body weight alone, without taking into account the metabolic background, cannot predict early enough how large this decrease will be. The observation of the levels of the ketone bodies opens such possibility and allows to capture the patients at risk of the greatest decrease in the body weight (Figs. 3, 4 and 6d). Except measuring the body weight and height there are no additional criterions (e.g. lean body mass, body composition analysis, grip strength) used in a routine clinical practice. We believe that 3HB, an indicator of ketogenic amino acid degradation, could be a valuable parameter of the patient’s nutritional status, because it allows for more effective identification of the patients at risk—the serum proteins, often used as the biomarkers of nutritional status, reflect rather inflammatory processes that accelerate nutritional depletion than a nutritional status itself (Bharadwaj et al. 2016). Oral mucositis induced by radiation is an inevitable, but transient inflammatory side-effect of radiotherapy. Also chemotherapy may induce inflammatory events (Vyas et al. 2014). C-reactive protein (CRP), a protein that rises in the blood with inflammation, revealed to be a useful laboratory parameter in monitoring of treatment toxicity in HNC patients undergoing RT/CHRT (Chatterjee and Mukherjee 2013; Wygoda et al. 2016). Prealbumin and albumin are negative acute phase proteins: they decrease in infection, inflammation, and trauma (Ritchie et al. 1999). 3HB, in addition to its activity as an energetic metabolite, performs cellular signaling functions and can suppress inflammation (Youm et al. 2015; Bae et al. 2016; Newman and Verdin 2017).

Multivariate projection methods (e.g. PCA, PLS-DA, OPLS-DA) allow to track the temporal changes in the metabolism of the individual patients (Abd Rahman et al. 2013; Correia et al. 2015; Guo et al. 2014). Such PCA based time trajectories were also used to identify the metabolic perturbations in the biofluids from mice during different phases of radiation sickness (Khan et al. 2011). The models, when built on a suitably large and representative group, provide an overview of the global metabolic changes occurring in serum during the treatment that can be useful for a quick detection of the patients at high risk of developing cachexia. In our study an exemplary time-dependent trajectory of the metabolic profiles for a chosen high risk patient (the most outlying patient in the PCA score plot, Fig. 3a) was constructed by PCA using the JRES NMR spectra. Table 1 collects the values of 3HB as well as other clinical nutritional status parameters gathered for this particular patient during the treatment course. This patient’s on-treatment changes in the serum concentrations of the ketone bodies and the weight loss (22.9%) are the highest in the whole group. The patient suffered due to the ARS symptoms (leading to dysphagia). The supportive treatment (local, oral, intravenous pharmacotherapy) was given to the patient after the 4th week of RT/CHRT (spectrum no. 6 in Fig. 3a) when the weight loss exceeded 10% (11.2%), the level of 3HB increased ~ 250 times (vs. the initial value) and PreALB fell below the reference values—it is worth noting that the ALB and BMI values were still in the normal range (Table 1). The supportive nutritional treatment resulted in a temporal decrease of 3HB—and the points (no. 7 and 8) representing the NMR spectra acquired after the onset of this additional care moved towards the central area of the plot (Fig. 3a); also CRP decreased and PreALB increased. However, despite the supportive therapy, the patient developed cachexia, with a weight loss progressing up to 22.9% at the end of RT/CHRT (Table 1)—after a temporary reduction, the levels of 3HB and CRP started to rise again (the patient’s NMR spectra no. 9 and 10 in Fig. 3a fall into the outlier area, which is due to the increased ketone bodies, Fig. 3b) and decrease in PreALB (Table 1). We can only assume that an earlier introduction of a supportive therapy (i.e. in the second week of RT/CHRT, as suggests the NMR spectrum no. 4, Fig. 3a) would improve the control over the pathologic metabolic processes. Such monitoring will be tested in our future studies.

The OPLS-DA results (the 1D ^1^H JRES NMR serum spectra) make it possible to identify the course of the metabolic changes associated with the weight loss during the treatment (Fig. 4 as well as Table 3 showing the statistical importance of the detected metabolic alterations). In Fig. 4 the entire database of the serum 1D ^1^H JRES NMR spectra (for all tumor sites, including the patients with the missing body weight measurements) is shown. The first symptoms of upcoming weight loss (Fig. 4b) are the reduction of lipids, alanine as well as the increase of lactate and N-acetyl-glycoprotein (NAG). The lipid signals are strongly attenuated in the 1D ^1^H JRES NMR spectra, however the analysis of the DIFF spectra (data not shown) did not confirm a correlation between the reduction of serum lipids and the patients’ weight loss. Instead, the observed decrease of the lipid signals was found to correlate with the doses of radiation received by the patients, which is in agreement with the previous observations found in radiotherapy treated head and neck (Srivastava et al. 2014; Jelonek et al. 2014) and breast cancer patients (Shaikh et al. 2017). Lowering of the serum lipids can be explained as being due to a tumor response to the treatment (Shaikh et al. 2017). The detailed inspection of the lipid peaks (at 0.9 to 1.3 ppm) of the CPMG NMR spectra (Fig. 2) also shows the effect of a progressive decrease in the serum lipid levels during the therapy.

Table 3 reveals no statistically significant differences in the glucose levels between the studied groups. The glucose levels were found to generally lower during the RT/CHRT course, however in the higher percentage weight loss groups this drop is more pronounced. There is a number of works reporting fluctuations in the blood glucose level in no-diabetes, however the complex network of self-regulating systems aimed at maintaining blood glucose within a relatively tight equilibrium is rather efficient (Weissman and Binah 2014). In our previous work a decrease of glucose was found to correlate with severity of ARS, but also without a statistical significance (Boguszewicz et al. 2016).

Among the important molecular events identified in Fig. 4 and verified statistically in Table 3 there is an increase in N-acetyl containing glycoproteins (NAGs). NAGs, mainly N-acetylglucosamine and N-acetylneuramic acid, strongly contribute to the weight loss classes separation. They are acute phase proteins with anti-inflammatory properties and are expressed more during inflammation and immune responses (Bell et al. 1987; Torri et al. 1999)—that is why NAG is called an NMR marker of inflammation. When studying the head and neck cancer treatment toxicity (Boguszewicz et al. 2016) we found that NAG was significantly positively correlated with CRP. However, NAG and CRP most likely capture the different aspects of the inflammatory response, as their half-lives differ (Connelly et al. 2017). We identified elevated NAG as well as decreased alanine as some of the characteristic features of high ARS in HNSCC patients, but alanine is also strongly connected with energy metabolism (Boguszewicz et al. 2016). According to Table 3, a statistically significant increase of NAG and a decrease of alanine correlate with the degree of weight loss. Inflammation is a local and systemic response to radiation injury and precedes occurrence of oral mucositis (Lalla et al. 2008); in turn, acute mucositis can worsen dysphagia (Bonomi et al. 2012; Schindler et al. 2015). It has been postulated that cachexia is an integrated physiological response of substrate mobilization driven by inflammation (Aoyagi et al. 2015), furthermore, inflammatory markers are positively associated with fatigue (Xiao et al. 2016), one of the features of cachexia (Stewart et al. 2006).

The next phase in the anti-clockwise transition from the lowest to the highest weight loss seen in Fig. 4 involves a massive secretion of the ketone bodies as well as increase of the signals arising from choline containing compounds—both are statistically significant in discrimination between the particular groups representing the weight loss (Table 3). Such large increase in the concentration of the ketone bodies is most likely associated with starvation caused by a severity of ARS, as discussed in the analysis of the time trajectories. 3HB is a signaling molecule in starvation response, e.g. it has a key role in the regulation of energy homeostasis during food-shortage, it is involved (together with AceAce) in the regulation of insulin signaling, it improves neuronal survival during nutrient deprivation in a mechanism presumably dependent on autophagy as well as regulates inflammation response (Rojas-Morales et al. 2016). A complex function of 3HB was also suggested by Ros-Mazurczyk et al. (2017). They detected increased 3HB in HNSCC patients using mass spectrometry and suggested that 3HB may be a kind of molecular marker of radiation response. However, as reveals from our study, though an increase in 3HB is observed in almost all patients, it is very low in those who did not show a significant decrease in the body weight, even if they receive intensified treatment due to a high staged tumor. Thus, radiation seems to has much less impact (if at all) on the 3HB increase than malnutrition. Some support for this supposition comes from the studies of the of breast cancer cells (Bartmann et al. 2018): the authors failed to find any influence of 3-hydroxybutyrate on the sensitivity to chemotherapy or ionizing radiation in the cell lines tested.

Choline, another metabolite seen in this transition phase (Fig. 4), is a quaternary amine synthesized in the liver, but its main sources are dietary intake and liberation from its reservoir within the membrane phosphatides of all mammalian cells (Wurtman et al. 2009). It is an important intermediate of phospholipid metabolism and a precursor in the synthesis of acetylcholine and phosphocholine, an essential component in membrane structure. Prolonged fasting as well as removal of all choline-containing foods from the diet reduces blood choline levels (Wurtman et al. 2009). The augmented utilization of phosphocholine for repairing the damaged cells/organelles under severe oxidative and systemic inflammatory condition should also lead to decrease in total Cho. On the other hand, in hypoxic states choline is released from hypoxic tissue into the plasma, thus increasing the basal circulating levels (Storm et al. 2013). The efficient source of choline is phosphatidylcholine hydrolysis (Fagone and Jackowski 2013). Inflammatory, even moderate hypoxia and other membrane-damaging stimuli—like those induced by radio- and chemo-therapy—trigger a cell stress pathway that is propagated by the hydrolysis of membrane phospholipids (Epstein 2003). Our observations indicate that the increase in the serum choline levels during the RT/CHT, taking place despite starvation and increased choline demand caused by the intensified repair processes (regeneration of cell membranes requires reduction of choline to phosphocholine) (Xu et al. 2017), may be a net effect of all the mentioned processes. As reported in our previous work (Boguszewicz et al. 2016), in a week after RT/CHRT completion the choline levels lower markedly which correlate with the intensiveness of ARS. When combining the after-treatment results with the current on-treatment data, it may be supposed that the decrease in blood choline after the therapy completion may reflect the increased choline uptake due to the prevalence of the cell membrane regeneration processes.

The final metabolites that also contribute to the weight loss class separation in the scores plot in Fig. 4 are propylene glycol and acetate. Propylene glycol seems to be an exogenous contaminant, as it is widely used as a solvent in many pharmaceuticals (Psychogios et al. 2011). Considering the weight loss as a result of an intensified ARS, the increase in the signal from propylene glycol in the patients with > 10% weight loss most probably results from the increased drug supplementation aimed at alleviation of the ARS symptoms, e.g. lorazepam or diazepam. Acetate as well as the ketone bodies result from the beta-oxidation of adipose tissue fatty acids under the starved condition (Yamashita et al. 2001). However, the production of acetate is several times smaller than of e.g. 3HB (Yamashita et al. 2001). In our previous study we observed the elevated acetate levels in the high ARS patients which correlated with their weight loss, but with no statistical significance (Boguszewicz et al. 2016). Thus, those findings are in accord with the current results.

Summarizing, the metabolic signatures obtained in HNSCC patients’ blood serum using high resolution ^1^H NMR show significant treatment-related alterations reflecting the severity of ARS and, consequently, the severity of weight loss. In some instances the disturbances are so pronounced that manifest themselves in the presence of the outliers—the cases that are different from the majority of data. The outliers affect the multivariate projection models and data clustering as well as significantly impede the detection of any subtle changes. Most data mining methods discard outliers as noise or exceptions, however the detection of the latter reveals to be very useful in the field of medical applications. Outlier-based monitoring and alerting has potential to complement the use of the clinical knowledge-based patient alerting systems. From the presented analysis of the metabolic phenotyping of blood plasma serum in HNSCC patients treated with RT/CHRT, a possibility emerges for using the outlying points—corresponding mainly to the high levels of 3-hydroxybutyrate—to identify the patients at risk of cachexia. 3HB—as the fast and sensitive biomarker of malnutrition or cachexia—should be treated still with caution. It means that the inclusion of supportive treatment (e.g. high-calorie nutrition, intravenous rehydration and nutrition and/or parenteral nutrition as well as local, oral and/or intravenous pharmacotherapy) will lower the 3HB level promptly (though temporally), however, it may not stop the ongoing cachectic processes and the progression of weight loss. The 3HB levels are also increased in diabetics and when using a ketogenic diet—such patients require special attention in analyzing the 3HB changes. Nevertheless, the metabolomic real-time monitoring of the serum ketone bodies—as a fast, simple and cheap method—seems to be very convenient in delivering clinically important information on treatment response and in complementing current risk models with additional parameters.

## Conclusion

In conclusion, this paper validates ^1^H NMR metabolic phenotyping of blood serum as a useful tool to identify high-risk HNSCC patients during the RT/CHRT treatment. The outlier detection in the multivariate projection models has the advantage over the simple laboratory tests alone, as it assesses more directly the complex interactions between treatment, tumor and patient.

Our findings indicate that metabolic alterations, characteristic for malnutrition or cachexia, can already be detected at an early stage. The NMR biomarkers of malnutrition or cachexia seem to be clinically useful and, consequently, it might be anticipated that the serum metabolomic screening and careful identification of the patients at risk of malnutrition or cachexia may lead to better outcomes and treatments. Thus, combining the molecular and clinical information provides a better insight into the patient’s status.

## Electronic supplementary material

Below is the link to the electronic supplementary material.

Supplementary material 1 (DOCX 18 kb)
