# Supplementary material for: NMR-based metabolomics in real-time monitoring of treatment induced toxicity and cachexia in head and neck cancer: a method for early detection of high risk patients
Source: Metabolomics. 2019 Aug 16;15(8):110. doi: 10.1007/s11306-019-1576-4 (PMC6697714; doi:10.1007/s11306-019-1576-4)
Supplement: Supplementary file 1 — Supplementary material 1 (DOCX 18 kb) [file 11306_2019_1576_MOESM1_ESM.docx]

**NMR-based metabolomics in real-time monitoring of treatment induced toxicity and cachexia in head and neck cancer – a method for early detection of high risk patients**

Ł. Boguszewicz ^1^, A. Bieleń ^2^, J. Mrochem-Kwarciak ^3^, A. Skorupa ^1^, M. Ciszek ^1^, A. Heyda ^2^, A. Wygoda ^2^, A. Kotylak ^2^, K. Składowski ^2^, M. Sokół ^1^

^1^ *Department of Medical Physics, Maria Sklodowska-Curie Memorial Cancer Center and Institute of Oncology, Gliwice Branch, Poland*.

^2^ *I Radiotherapy Clinic, Maria Sklodowska-Curie Memorial Cancer Center and Institute of Oncology, Gliwice Branch, Poland*.

^3^ *Analytics and Clinical Biochemistry Department, Maria Sklodowska-Curie Memorial Cancer Center and Institute of Oncology, Gliwice Branch, Poland*.

*Maria Sklodowska-Curie Memorial Cancer Center and Institute of Oncology, Gliwice Branch*

*Street: Wybrzeze Armii Krajowej 15,*

*44-101 Gliwice, Poland.*

Corresponding author:

Lukasz Boguszewicz

Phone: +48322788047

Fax: +48322313512

[Lukasz.boguszewicz@io.gliwice.pl](mailto:Lukasz.boguszewicz@io.gliwice.pl)

#### Acknowledgments

The work has been funded by National Science Centre grant 2015/17/B/NZ5/01387 **Patients**

The studied group consisted of 170 HNSCC patients treated oncologically in the 1st Radiation and Clinical Oncology Department of MSC Memorial Institute in Gliwice. Cancer was diagnosed in fifteen anatomical sub-regions of the head and neck, with distinction of four major sites: nasopharynx (11 patients), oropharynx (50 patients), hypopharynx (22 patients), larynx (77 patients) and other (10 patients).

Table S1. Detailed description of the studied group with distinction of anatomical sub-regions affected by tumor.

| ICD10 | Anatomic  sub-regions | Major anatomic sites* | T1 | T1N+ | T2 | T2N+ | T3 | T3N+ | T4 | T4N+ | T0N+ | Total |
| --- | --- | --- | --- | --- | --- | --- | --- | --- | --- | --- | --- | --- |
| C01 | Base of tongue | Oropharynx | 1 | 1 | 1 | 1 |  | 2 | 1 | 2 |  | 50 |
| C09 | Tonsil |  | 1 | 1 | 3 | 4 |  | 9 | 1 | 5 | 2 |  |
| C10 | Oropharynx |  |  |  |  | 2 | 2 | 6 |  | 6 |  |  |
| C11 | Nasopharynx | Nasopharynx |  | 2 |  | 4 |  | 3 |  | 1 | 1 | 11 |
| C12 | Pyriform sinus | Hypopharynx |  |  | 1 | 1 |  |  | 1 |  |  | 22 |
| C13 | Hypopharynx |  |  |  |  | 6 |  | 5 | 1 | 7 |  |  |
| C32 | Larynx | Larynx (low stage) | 13 |  | 24 |  |  |  |  |  |  | 37 |
| C32 | Larynx | Larynx (high stage) |  |  |  | 8 | 17 | 6 | 4 | 5 |  | 40 |
| C02 | Other and unspecified parts of tongue | Other |  |  |  |  |  | 1 |  |  |  | 10 |
| C04 | Floor of mouth |  |  |  | 1 |  |  |  |  |  |  |  |
| C05 | Palate |  |  |  |  |  |  |  |  | 1 |  |  |
| C06 | Other and unspecified parts of mouth |  |  |  | 1 |  |  |  |  |  |  |  |
| C30 | Nasal cavity and middle ear |  |  |  | 1 |  |  |  |  |  |  |  |
| C31 | Accessory sinuses |  |  |  |  |  |  |  | 1 |  |  |  |
| C77 | Lymph nodes |  |  |  |  |  |  |  |  |  | 1 |  |
| C80 | Without specification of site |  |  |  |  |  |  |  |  |  | 3 |  |
| Number of patients with particular tumor stage | | | 15 | 4 | 31 | 26 | 19 | 32 | 9 | 27 | 7 | 170/  100% |
| Percentage distribution of primary tumor stage | | | 11.2% | | 33.5% | | 30% | | 21.2% | | 4.1% |  |

* as listed in publication

**NMR pulse sequences parameters**

Table S2. Pulse sequence parameters.

| Pulse program | **NOESYGPPR1D** | **CPMGPR1D** | **LEDBPGPPR2S1D** | **JRESGPPRQF** |
| --- | --- | --- | --- | --- |
| **TD** | 65536 | 65536 | 65536 | 8192 |
| **SW** [ppm] | 30 | 20 | 30 | 16.62 |
| **AQ** [sec] | 2.73 | 4.09 | 2.73 | 0.62 |
| **D1** [sec] | 4 | 4 | 4 | 2 |
| **D8** [sec] | 0.01 | - | - | - |
| **D16** [sec] | - | - | 0.0002 | 0.0002 |
| **D20** [sec] | - | 0.0003 | 0.12 | - |
| **D21** [sec] | - | - | 0.005 | - |
| **DS** | 4 | 4 | 4 | 16 |
| **L4** | - | 126 | - | - |
| **NS** | 32 | 64 | 64 | 1 |
| **DELTA1** [sec] | - | - | 0.11572488 | - |
| **DELTA2** [sec] | - | - | 0.004172 | - |
